# Proteomic analysis of integrin‐associated complexes from mesenchymal stem cells

**DOI:** 10.1002/prca.201500033

**Published:** 2015-09-17

**Authors:** Jila N. Ajeian, Edward R. Horton, Pablo Astudillo, Adam Byron, Janet A. Askari, Angélique Millon‐Frémillon, David Knight, Susan J. Kimber, Martin J. Humphries, Jonathan D. Humphries

**Affiliations:** ^1^ Wellcome Trust Centre for Cell‐Matrix ResearchFaculty of Life SciencesUniversity of ManchesterManchesterUK; ^2^ Biological Mass Spectrometry Core FacilityFaculty of Life SciencesUniversity of ManchesterManchesterUK; ^3^ North West Embryonic Stem Cell CentreFaculty of Life SciencesUniversity of ManchesterManchesterUK

**Keywords:** Extracellular matrix, Integrin, LIM domain, Mechanotransduction, Mesenchymal stem cell

## Abstract

**Purpose:**

Multipotent mesenchymal stem cells (MSCs) have the capability to differentiate down adipocyte, osteocyte and chondrocyte lineages and as such offer a range of potential therapeutic applications. The composition and stiffness of the extracellular matrix (ECM) environment that surrounds cells dictates their transcriptional programme, thereby affecting stem cell lineage decision‐making. Cells sense force via linkages between themselves and their microenvironment, and this is transmitted by integrin receptors and associated adhesion signalling complexes. To identify regulators of MSC force sensing, we sought to catalogue MSC integrin‐associated adhesion complex composition.

**Experimental design:**

Adhesion complexes formed by MSCs plated on the ECM ligand fibronectin were isolated and characterised by MS. Identified proteins were interrogated by comparison to a literature‐based reference set of cell adhesion‐related components and using ontological and protein–protein interaction network analyses.

**Results:**

Adhesion complex‐specific proteins in MSCs were identified that comprised predominantly cell adhesion‐related adaptors and actin cytoskeleton regulators. Furthermore, LIM domain‐containing proteins in MSC adhesion complexes were highlighted, which may act as force‐sensing components.

**Conclusion and clinical relevance:**

These data provide a valuable resource of information regarding the molecular connections that link integrins and adhesion signalling in MSCs, and as such may present novel opportunities for therapeutic intervention.

**Abbreviations**
ECM: extracellular matrix
FN: fibronectin
LIM domain: LIN‐11, Isl1, and MEC‐3 domain
MSC: mesenchymal stem cell
PDL: poly‐d‐Lysine

**Clinical Relevance**

The use of mesenchymal stem cells (MSCs) for tissue engineering and regenerative medicine applications is attractive, in part due to their ability to differentiate into multiple cell types and their ease of expansion in vitro. MSC growth and differentiation are influenced by the extracellular environment, which is sensed by integrin cell‐surface receptors binding to extracellular matrix (ECM) components. This binding allows the formation of intracellular protein complexes that signal to determine specific cellular outcomes in response to different environmental cues. A more detailed knowledge of how MSCs sense the mechanical, compositional and topological features of the ECM via integrins and their associated proteins will aid our understanding of the regulation of MSC growth and differentiation, and in turn will benefit clinical applications of these cells.

Multipotent mesenchymal stem cells (MSCs) have the capacity to differentiate into multiple mesenchymal lineages 1 and to provide beneficial immunomodulatory factors. As such MSCs have attracted much attention with respect to their potential as therapeutic agents for tissue engineering and regenerative medicine applications 2, 3. Many tissues and cell types have been demonstrated to respond to the stiffness of their local extracellular matrix (ECM) environments by means of mechanosensitive signalling pathways that act via transcriptional reprogramming to impact on normal development, wound healing and diseases such as fibrotic disorders 4, 5. The extracellular environment is also a key driver of MSC differentiation, which is regulated by both the composition and the mechanical properties of the ECM that surrounds cells and tissues 6, 7, 8. The mechanosensitive regulation of MSC cell fate is transmitted through RhoA and ROCK to the actin cytoskeleton, which controls the nuclear and cytoplasmic localisation of the transcriptional co‐activators YAP and TAZ to regulate gene expression, resulting in MSC differentiation. In this way, ECM stiffness dictates MSC differentiation with stiff (bone‐like) substrates tending to induce osteoblasts, intermediate stiffness substrates leading to myoblasts and soft substrates leading to neurons or adipocytes 9, 10.

Integrins are a family of cell‐surface ECM receptors that mediate signalling across the plasma membrane from the extracellular environment to the actin cytoskeleton 11. Integrin‐ECM engagement nucleates the formation of a dynamic, intracellular, membrane‐proximal complex of proteins that links the ECM to the actin cytoskeleton 12. Integrins and their associated adhesion complex components (the composition of which has been termed the adhesome) are therefore ideally placed to relay mechanosensitive cell‐fate decisions in a variety of cell types including MSCs 13, 14. We hypothesised that a detailed understanding of the composition of adhesion complexes formed in MSCs upon integrin‐ECM ligation would improve our understanding of how the ECM and mechanosensitive signalling platforms are established and orchestrate cell fate decision making. We therefore isolated adhesion complexes from MSCs and catalogued their components by LC‐MS/MS using recently described approaches 15, 16, 17, 18. The information gained from our approach will benefit regenerative medicine and tissue engineering approaches that use MSCs.

Adhesion complex formation in human bone marrow‐derived MSCs (Lonza Bioscience) spread on fibronectin (FN) was confirmed by immunofluorescence staining for the well‐defined adhesion complex components vinculin, integrin‐linked kinase and active integrin β1 (Fig. 1A). Dishes coated with poly‐d‐Lysine (PDL) were used as a control substrate that allowed cell spreading but did not support engagement of integrin and the formation of integrin‐associated adhesion complexes (Fig. 1A). Adhesion complexes were then isolated from MSCs spread on FN and PDL using a published method (Fig. 1B) 18. Western blotting of isolated complexes confirmed the specific recruitment of the integrin subunits β1 and α5, along with talin, a well‐characterised integrin‐binding cytoskeletal adaptor protein 14 (Fig. 1C) in cells spread on FN compared to those on PDL. These data demonstrated that MSCs formed adhesion complexes upon adhesion to FN which were amenable to isolation by published biochemical approaches.

**Figure 1:**
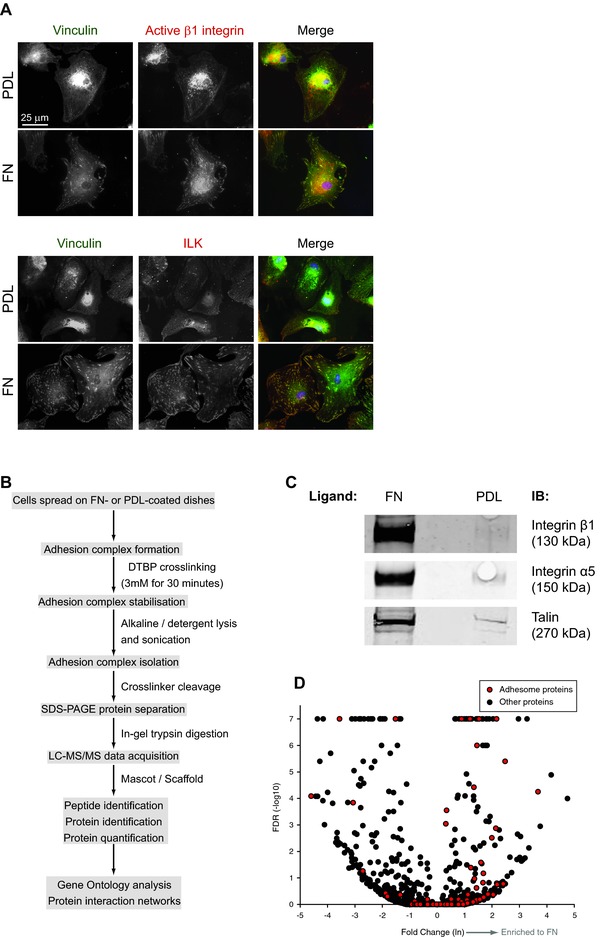
Isolation and proteomic analysis of adhesion complexes from MSCs. Human bone marrow‐derived MSCs obtained from a 22‐year‐old female donor (Lonza BioScience) were isolated from human tissue with informed consent and were validated for the positive expression of CD105, CD166, CD29 and CD44 and for the negative expression of CD14, CD34 and CD45 by the supplier. Independent validation of positive expression of CD105 and negative expression of CD14 were performed by flow cytometry before use (data not shown). (A) Immunofluorescence images of MSCs spread on 10 μg/mL FN or PDL (Sigma‐Aldrich) for 3 h, fixed with 4% (w/v) paraformaldehyde, permeabilised with 0.05% (w/v) Triton X‐100 and visualised with antibodies directed against vinculin (hVIN‐1; Sigma‐Aldrich), ILK (EPR1592; Abcam) or active β1 integrin (9EG7; provided by D. Vestweber, Max Planck Institute for Molecular Biomedicine, Germany). Images were collected on an Olympus BX51 upright microscope using a 20×/0.50 Plan Fln objective, captured using a Coolsnap HQ camera (Photometrics) through MetaVue software (Molecular Devices) and processed using ImageJ (http://rsb.info.nih.gov/ij). (B) Workflow of adhesion complex isolation and MS analysis. The analyses were performed in biological triplicate on either Orbitrap elite or Velos Pro systems (Thermo Fisher Scientific). (C) Adhesion complexes isolated from MSCs were subjected to SDS‐PAGE and Western blotting for β1 integrin (JB1A; provided by J. A. Wilkins, University of Manitoba, Canada), α5 integrin (H‐104; Santa Cruz Biotechnology) or talin (C‐20; Santa Cruz Biotechnology). (D) Volcano plot displaying all proteins (black circles) or adhesome proteins (red circles) identified by MS from adhesion complexes isolated from MSCs. Statistics were determined by QSpec analysis (http://www.nesvilab.org/qspec.php/; [37]). Values for FDR(‐log10) of 7 represent calculated FDR values of 0 in Qspec.

For proteomic analysis of integrin‐associated complexes, MSCs were spread on FN and the control ligand PDL and the isolation procedure performed in triplicate. Samples were analysed by LC‐MS/MS. In total, from all conditions, MS analyses identified 1352 proteins with an FDR < 0.01% and a minimum of two unique peptides per protein (Supporting Information). Using spectral counts as a measure of protein abundance, and a subtractive approach utilising control PDL isolations, 475 proteins were classified as specifically enriched to protein complexes isolated from cells spread on FN over PDL (≥twofold FN:PDL normalised spectral count ratios; Supporting Information). Further analysis of the dataset highlighted 86 proteins as statistically enriched to FN compared to PDL (FDR<1%), including the integrin α5 and β1 subunits along with several well‐characterised adhesion complex components (Supporting Information). The current literature‐curated adhesome inventory reported 232 proteins 12 and our analyses identified 74 of these proteins (32%) from all conditions (Fig. 1D; Supporting Information) 12. Adhesome components have been characterised as either intrinsic components, which localise directly to adhesion complexes, or associated components, which are effectors of intrinsic molecules 19. Importantly, 47 of the 74 adhesome proteins (38 intrinsic and 9 associated components, respectively; Supporting Information) were determined to be enriched to complexes isolated from cells spread on FN compared to PDL, a comparable number to previous analyses of this kind 20, 21. Therefore, these data reveal the subset of the adhesome that is found in adhesion complexes in MSCs. To gain an unbiased, global perspective of the dataset, Gene Ontology (GO) enrichment analysis was performed on FN‐enriched proteins using the online bioinformatic tools available via the Database for Annotation, Visualization and Integrated Discovery (DAVID; http://david.abcc.ncifcrf.gov/home.jsp) 22. These analyses confirmed that proteins identified using this approach were enriched for terms relating to cell‐substrate junctions, cell adhesion, cell migration and connections to the actin cytoskeleton, as expected for adhesion complex proteins (Supporting Information).

To provide a simplified view of the molecular organisation of the isolated adhesion complexes in the context of known adhesion complex protein interactions, a network was constructed using the identified MSC adhesome components and organised according to their defined roles (Fig. 2) 19. This analysis highlighted that, while adhesome proteins were identified from a wide variety of functional groups, from an adhesome‐based perspective, the majority of FN‐enriched proteins were identified from the adaptor and actin regulator subgroups of the adhesome. Importantly, as for adhesion complexes from other cell types and potentially relating to cell fate determination, these findings are indicative of MSC adhesion complexes performing the dual roles of establishing connections to the actin cytoskeleton and acting to nucleate adaptor proteins as signalling hubs. In addition, MS analysis of isolated MSC adhesion complexes identified only a restricted set of integrin FN receptors that were enriched to FN compared to the control PDL (Fig. 2). To assess the repertoire of integrins on the cell surface of MSCs, flow cytometry was performed. In agreement with previous studies 23, these analyses revealed that MSCs express a number of integrin heterodimers that can bind a variety of ECM ligands 24, including FN (α5β1, αVβ1 and αVβ3), collagen (α1β1 and α2β1) and laminin (α3β1 and α6β1) (data not shown). Investigation of the numbers of peptides identified for each integrin subunit revealed that the major integrin identified by MS in MSC adhesion complexes was α5β1 (Supporting Information). Therefore, these data indicate that, under the conditions assessed, MSCs primarily utilise α5β1 to engage FN, and therefore the associated protein identifications in this study are likely to be from adhesion complexes predominantly formed by integrin α5β1.

**Figure 2:**
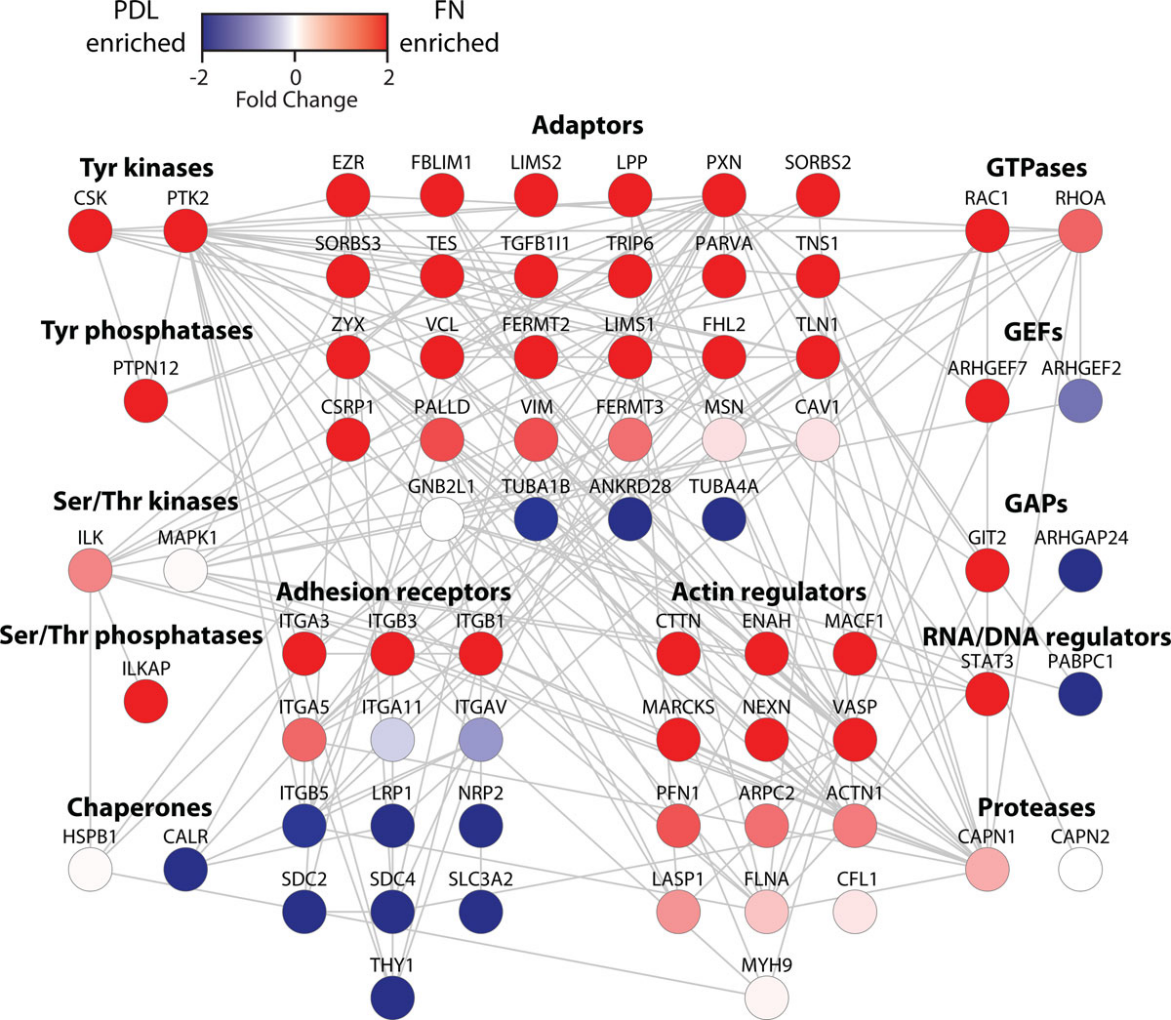
Protein–protein interaction network of adhesome components identified in MSC cells. Interaction network analysis of adhesome components isolated from FN or PDL‐induced adhesion complexes. Nodes (circles) represent identified proteins and are labelled with gene symbols, arranged according to their reported functional group 19 and coloured with respect to their enrichment to FN or PDL as defined by their normalised spectral count ratios. Edges (grey lines) indicate reported protein–protein interactions. Interaction network analysis was performed as previously described 17 using Cytoscape (version 2.8.3) and a merged human interactome comprising protein–protein interactions reported in the Protein Interaction Network Analysis platform, MatrixDB and the adhesome. Out of the 232 adhesome components, 74 were detected in the dataset and 47 of these were enriched ≥twofold to FN compared to PDL. PLEC and NRP1 did not map on to the interaction network database. GEF, guanine nucleotide exchange factor; GAP, GTPase‐activating protein.

Previous MS analyses of adhesion complexes isolated from fibroblasts have established that the LIN‐11, Isl1 and MEC‐3 (LIM) domain‐containing proteins are recruited to adhesion complexes via forces generated by the myosin II contractile machinery 15, 16. Similar forces have been shown to regulate the ECM elasticity‐dependent control of MSC cell fate via YAP/TAZ 9. Interestingly, GO enrichment analysis of proteins identified by MS from MSC adhesion complexes demonstrated a significant enrichment of LIM domain‐containing proteins (Supporting Information), as previously observed for adhesion complexes isolated from fibroblasts 15, 16. In total, 24 LIM domain proteins were identified in the dataset, 21 of which were at least twofold enriched to FN (Supporting Information). Visualisation of the identified LIM domain proteins in the context of their known interactions with proteins from the adhesome highlighted the potential interplay between LIM domain proteins in MSC adhesion complexes and the adhesome (Fig. 3A). As some LIM domain proteins convey mechanosensitive signals through adhesion complexes to the actin cytoskeleton 25, 26, we hypothesised that the LIM domain proteins identified in MSCs in this study may play roles in MSC cell fate determination and localise at different cellular locations in response to altered ECM stiffness. We therefore investigated the mechanosensitive localisation of PDLIM1, PDLIM5 and PDLIM7, which are LIM domain proteins identified in this study that are not currently categorised as adhesome components. MSC cells spread on a stiff substrate (glass) coated with FN formed prominent vinculin‐positive adhesion complex structures, a subset of which colocalised with, or closely to, clusters of PDLIM1 or PDLIM5 (Fig. 3B). Interestingly, PDLIM1, PDLIM5 and PDLIM7 displayed distinct subcellular localisation patterns in MSCs. For example, PDLIM7 localised away from the leading edge of cells, unlike PDLIM1 and PDLIM5. Moreover, MSCs spread on a soft substrate (12 kPa; Matrigen) formed fewer vinculin‐positive adhesion structures and displayed an altered localisation of PDLIM1, PDLIM5 and PDLIM7 (Fig. 3B). Importantly, the substrate elasticity used for these studies was in the range that resulted in a nuclear‐cytoplasmic redistribution of YAP/TAZ (data not shown). In brief, cells spread on glass or 12 kPa stiffness demonstrated nuclear YAP/TAZ, whereas cells on 0.5 kPa stiffness displayed cytoplasmic retention of YAP/TAZ, smaller spreading areas and did not form vinculin‐positive adhesion complexes. These data highlight that proteins identified from the MSC adhesion complex dataset, such as PDLIM1, PDLIM5 and PDLIM7, can display tension‐dependent relocalisation within cells and indicate that the dataset may be of benefit for future study by the wider research community.

**Figure 3:**
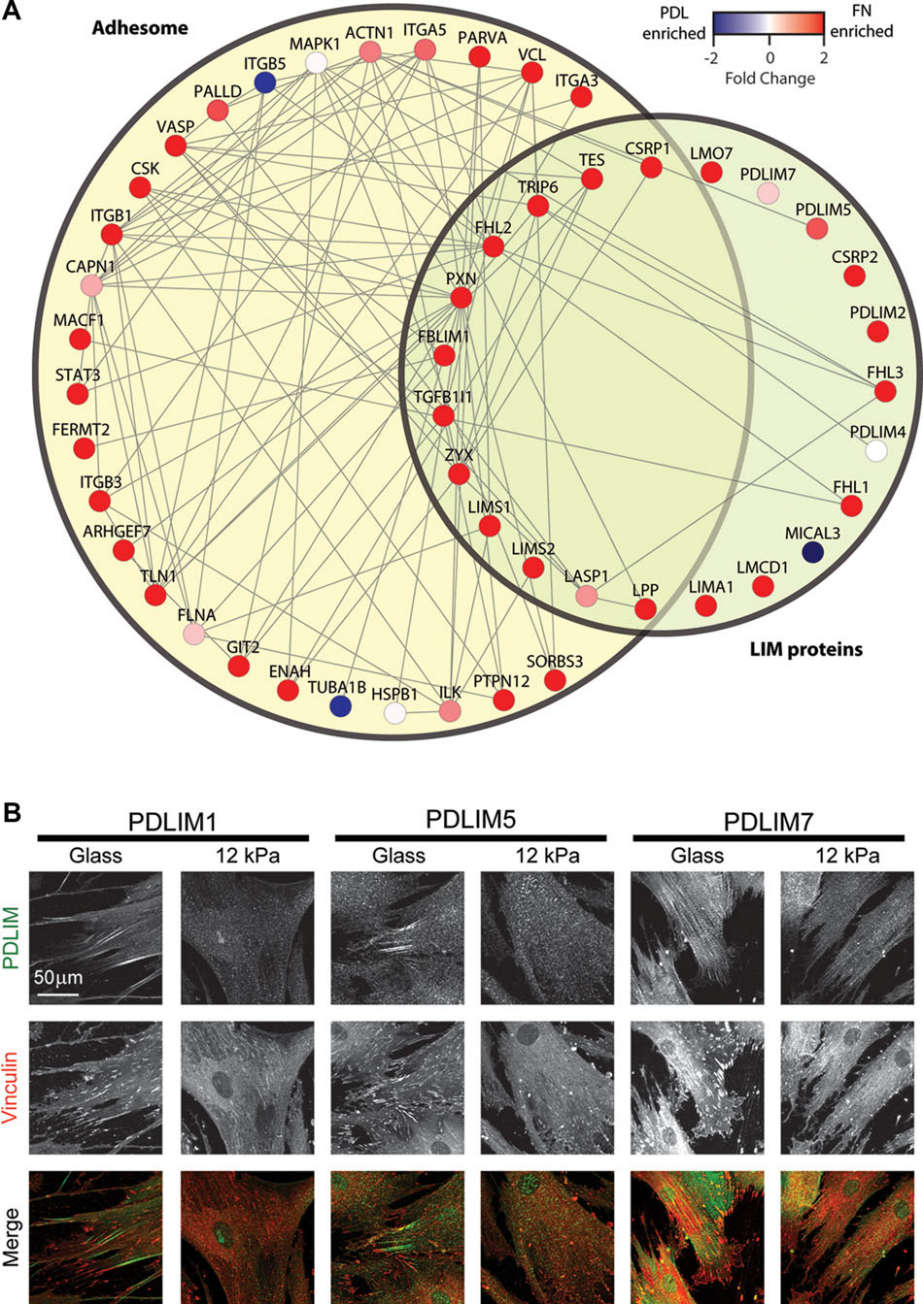
LIM domain proteins identified in adhesion complexes from MSCs. (A) Protein interaction network of all LIM proteins identified by MS and proteins with which they reportedly directly interact in the adhesome. The non‐adhesome LIM protein PDLIM1 did not map on to the interaction network database. (B) Immunofluorescence images of MSCs spread on glass or polyacrylamide‐coated coverslips with defined stiffness (12 kPa, Matrigen) previously coated with FN (20 μg/mL). MSCs were cultured for 24 h before fixation with 4% (w/v) paraformaldehyde and visualised using antibodies against PDLIM1 (ab64971, Abcam), PDLIM5 (E‐25 and JK‐3R, Santa Cruz Biotechnology) and PDLIM7 (ab86065, Abcam) and vinculin (hVIN‐1; Sigma‐Aldrich). Confocal microscopy images were collected on a Leica TCS SP5 AOBS upright confocal using a 20×/0.50 HCX Apo L water objective and processed using ImageJ.

In summary, we have enriched and isolated adhesion complex structures from MSCs spread on FN and catalogued their composition by LC‐MS/MS, revealing a subset of adhesome proteins found in MSCs that contained many potentially mechanoresponsive LIM domain‐containing proteins. Application of MSCs to cell therapy or tissue engineering requires an understanding of how to maintain and differentiate MSCs both in vitro and in vivo 27, 28. Moreover, ECM elasticity, ECM composition, integrins and their associated adhesion complex components are now widely appreciated to play roles in MSC growth, differentiation and homing 14, 29, 30, 31. FN is a component of the ECM that has been shown to regulate stem cell biology in a variety of systems including MSC cell fate decisions 32, 33, 34, 35. Therefore, we suggest that the proteins identified in the present study will inform strategies to utilise MSCs in basic science and for therapeutic applications. Future studies could be directed to further strengthen such findings by repeating these analyses and performing comparison of adhesion complex isolations from MSCs on soft versus stiff ECM substrates.

## Supporting information

As a service to our authors and readers, this journal provides supporting information supplied by the authors. Such materials are peer reviewed and may be re‐organized for online delivery, but are not copy‐edited or typeset. Technical support issues arising from supporting information (other than missing files) should be addressed to the authors.

Supplementary data: General Sample and Data Analysis Statistics
